# Exploring the Impact of Preoperative Laboratory Values on Short-Term Outcomes in Complex Carpal Tunnel Decompression Surgery

**DOI:** 10.1155/aort/8494043

**Published:** 2025-01-22

**Authors:** Anitesh Bajaj, Rushmin Khazanchi, Rohan M. Shah, Joshua P. Weissman, Nishanth S. Sadagopan, Arun K. Gosain

**Affiliations:** Division of Plastic Surgery, Lurie Children's Hospital of the Northwestern University Feinberg School of Medicine, Chicago, Illinois, USA

## Abstract

**Background:** The present study analyzes the effects of preoperative serum albumin, hematocrit, and creatinine on postoperative outcomes in patients undergoing carpal tunnel decompression surgery.

**Methods:** The American College of Surgeons National Quality Improvement Program (NSQIP) database was queried from 2011 to 2020. Albumin, hematocrit, and creatinine were collected for each patient, alongside covariates. Outcomes included 30-day medical complications, 30-day wound complications, return to the operating room, nonhome discharge, and extended postoperative length of stay. Bivariate *t*-tests and multivariate logistic regressions were conducted. For any outcome-laboratory value pairs with significance on regression, area under the receiver operating characteristic curves (AUC) were constructed.

**Results:** A total of 1440 patients with albumin, 3138 patients with hematocrit, and 3159 patients with creatinine levels were identified. Increased serum albumin was associated with lower odds of medical complications (aOR: 0.479, *p*=0.035). An overall cohort cutoff of ≤ 3.5 g/dL (AUC: 0.79, *p* < 0.001) was predictive of medical complications. On multivariate logistic regression, increased hematocrit reduced the odds of medical complications (aOR: 0.889, *p* < 0.001). Predictive hematocrit cutoffs of ≤ 39.7% (AUC: 0.77, *p* < 0.001) and ≤ 36.6% (AUC: 0.74, *p* < 0.001) were identified for medical complications amongst male and female patients, respectively. Similarly, increased serum creatinine was associated with greater odds of medical complications (aOR: 1.684, *p*=0.006). Creatinine cutoffs of ≥ 1.2 mg/dL (AUC: 0.58, *p*=0.033) and ≥ 1.0 mg/dL (AUC: 0.59, *p*=0.039) were identified for medical complications amongst male and female patients, respectively.

**Conclusions:** Multiple preoperative serum values were predictive of postoperative medical complications, and laboratory value thresholds were identified in this carpal tunnel decompression cohort to aid in risk stratification.

## 1. Introduction

Carpal tunnel syndrome (CTS) is a common condition that often causes pain, discomfort, and paresthesia in the hand or forearm. An estimated 1%–5% of people worldwide suffer from CTS, and patients with the condition can have highly variable clinical presentation and demographics [1]. Given that carpal tunnel decompression surgery is generally a nonemergent procedure, surgeons and patient care teams have the ability to optimize patient profiles prior to surgery in order to reduce postoperative complications. Previous research has assessed the safety and risk profile for patients opting to undergo carpal tunnel release [2, 3]. Some of the most identified complications following surgery include infection, fluid collection, hemorrhage, median nerve damage, chronic pain, and atrophy of the thenar muscles, among others [4, 5]. Some studies have analyzed predictors for complications in carpal tunnel surgery; however, very few have evaluated whether preoperative laboratory (lab) values may inform postoperative expectations [6, 7].

Serum albumin, creatinine, and hematocrit have been identified as predictors of increased morbidity and wound complications in surgical patients [8–11]. Although these metrics have been studied in other fields, the current literature has yet to analyze these variables in carpal tunnel patients. Therefore, the present study sought to identify whether preoperative albumin, serum creatinine, and hematocrit were associated with increased postoperative morbidity in patients undergoing carpal tunnel release. Elucidating risk factors may help guide preoperative risk management, influence shared decision-making, and frame patient expectations in this highly prevalent condition. This study aims to investigate the capability of preoperative lab values (hematocrit, albumin, and creatinine) in predicting short-term outcomes in complex carpal tunnel surgery patients.

## 2. Methods

### 2.1. Data Source and Processing

The American College of Surgeons National Quality Improvement Program (NSQIP) database was queried for carpal tunnel decompression surgeries (CPT 29,848, 64,721) from 2011 to 2020 to conduct this national retrospective cohort study. The goal of this study was to assess complex carpal tunnel decompressions; therefore, the NSQIP was chosen because it captured both inpatient and outpatient surgeries. All data processing for the NSQIP data was conducted through the nsqipr R package. Per NSQIP guidelines, a surgical clinical reviewer is responsible for collecting data at each participating site, and inter-rater reliability audits are intermittently conducted for quality control. Although not all cases for a given CPT code are recorded at specific sites, systematic sampling methods for each CPT code are leveraged to select cases which are recorded in the NSQIP database to ensure that cases have equal chances of being selected from each day of the week.

The latest recorded preoperative serum lab values within 90 days of surgery for hematocrit, creatinine, and albumin were collected for each patient, alongside relevant demographic and clinical covariates (Supporting Table 1). Outcomes included return to the operating room, nonhome discharge (discharge disposition that was not classified as a home or a facility that is considered a home), extended postoperative length of stay (eLOS) which is a measure of days from operation to discharge (75th percentile in our cohort), 30-day aggregate medical complications (occurrence of pneumonia, reintubation, pulmonary embolism, failure to wean, renal insufficiency, renal failure, urinary tract infection, stroke, cardiac arrest, myocardial infarction, bleeding, deep vein thrombosis, systemic sepsis, and/or shock), and 30-day aggregate wound complications (defined as superficial infection, wound infection, organ space infection, and/or wound dehiscence). Since not all patients had all preoperative lab values recorded within the preoperative window, each lab value was assessed in separate cohorts. Within each cohort, cases missing data for any demographic or clinical covariates included in Supporting Table 1 were excluded from the analysis set.

### 2.2. Statistical Analysis

Bivariate two-sided Welch's *t*-tests were assessed for each lab value to compare the average serum lab value between patients that did and did not experience each studied outcome. For any bivariate analyses that were significant, a multivariate logistic regression model was constructed with all clinical and demographic covariates included as control variables to assess the independent capability of the lab value in predicting the specified outcomes. Adjusted odds ratios (aORs) as well as significance values from a Wald's test were computed.

For any outcome-lab value pairs determined to be independent predictors of complications on regression, the area under the receiver operating characteristic curve (AUC) was computed, using its equivalence to the Wilcoxon rank sum statistic. The AUC measures the ability of each lab value in distinguishing between patients who did and did not experience the specified adverse outcomes, with an AUC of 0.5 suggesting a null predictive capability and 1.0 suggesting perfect predictive capability. Optimal predictive cutoff points were determined using Youden's index. Cutoff points were derived for the entire cohort in the case of albumin and separately for males and females when analyzing hematocrit and creatinine due to sex-specific reference ranges. Each cutoff point was further validated through an additional multivariate logistic regression that used the cutoff point to binarize the continuous serum lab values.

Significance was defined as *p* < 0.05. RStudio and a set of custom statistical scripts were used for all analyses.

## 3. Results

### 3.1. Albumin

A total of 1440 patients with serum albumin were analyzed in this study (Table 1). Within the albumin cohort, the average age was 59.6 years, 62.4% were female, and 48.8% were obese. The average albumin value was 4.0 g/dL (25^th^ percentile: 3.8 g/dL, median: 4.1 g/dL, 75^th^ percentile: 4.4 g/dL). The average postoperative length of stay was 1.0 day (75^th^ percentile: 1 day). Within 30 days postoperatively, 2.0% of patients underwent a return to the operating room, 4.2% of patients experienced a nonhome discharge, 1.9% of patients had a wound complication, 4.7% of patients experienced a medical complication, and 15.3% of patients had an eLOS.

**Figure 1:**
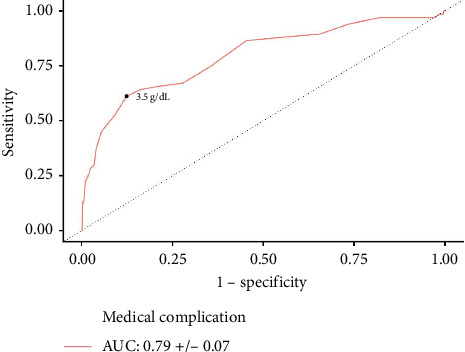
Albumin ROC curve for predicting medical complications. A predictive albumin threshold for medical complications was identified at ≤ 3.5 g/dL.

Bivariate analysis revealed lower albumin levels in patients who underwent a return to the operating room (3.71 g/dL vs. 4.03 g/dL, *p*=0.047), nonhome discharge (3.50 g/dL vs. 4.05 g/dL, *p* < 0.001), medical complication (3.34 g/dL vs. 4.06 g/dL, *p* < 0.001), and eLOS (3.67 g/dL vs. 4.09 g/dL, *p* < 0.001) (Table 2). Lower albumin levels were insignificant upon bivariate analysis for wound complications (*p* > 0.05). Upon multivariate analysis, which controlled for all demographic and clinical covariates, increases in albumin independently decreased the odds of medical complications (aOR: 0.479; 95% CI: 0.242–0.948; *p*=0.035). AUC analysis identified predictive cutoff values of ≤ 3.5 g/dL albumin for medical complications (AUC: 0.79 ± 0.065, *p* < 0.001) (Table 3) (Figure 1). Upon further testing of this cutoff in a logistic regression, patients with ≤ 3.5 g/dL albumin had increased odds of medical complications (aOR: 3.144; 95% CI: 1.366–7.236; *p*=0.007).

### 3.2. Hematocrit

In the hematocrit cohort, 3138 patients were analyzed. The average age was 58.7 years, 61.6% were female, and 47.7% were obese. The average hematocrit value was 40.3% (25^th^ percentile: 37.6%, median: 40.5%, 75^th^ percentile: 43.2%). The average postoperative length of stay was 0.9 day (75^th^ percentile: 1 day). When analyzing positive outcome frequencies, 1.9% of patients experienced a return to the operating room, 3.3% of patients had a nonhome discharge, 3.2% of patients experienced a wound complication, 1.8% of patients had a medical complication, and 13.4% of patients experienced an eLOS.

Bivariate analysis found significantly decreased hematocrit levels in patients who experienced nonhome discharge (36.16% vs. 40.39%, *p* < 0.001), medical complications (34.91% vs. 40.43%, *p* < 0.001), and eLOS (38.68% vs. 40.50%, *p* < 0.001). Lower hematocrit levels were insignificant upon bivariate analysis for return to the operating room and wound complications (*p* > 0.05). Multivariate analysis revealed that increases in hematocrit decreased the odds of medical complications (aOR: 0.889; 95% CI: 0.846–0.934; *p* < 0.001).

**Figure 2:**
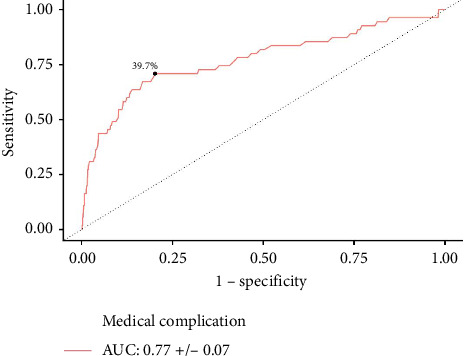
Hematocrit ROC curve for predicting medical complications in male patients. A predictive hematocrit threshold of ≤ 39.7% was identified for medical complications.

In males, AUC analysis revealed predictive hematocrit cutoffs of ≤ 39.7% for medical complications (AUC: 0.77 ± 0.074, *p* < 0.001) (Figure 2). This cutoff was tested in a multivariate regression, and male patients with ≤ 39.7% hematocrit had significantly increased odds of medical complications (aOR: 3.555; 95% CI: 1.351–9.351; *p*=0.010).

**Figure 3:**
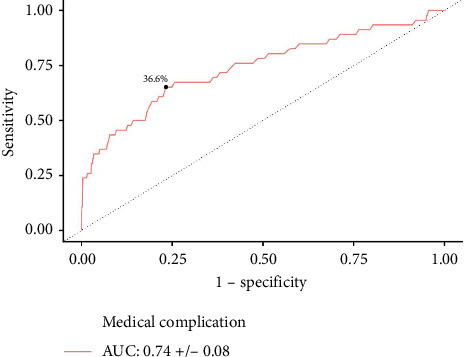
Hematocrit ROC curve for predicting medical complications in female patients. A predictive hematocrit threshold of ≤ 36.6% was identified for medical complications.

In females, AUC analysis found predictive hematocrit cutoffs of ≤ 36.6% for medical complications (AUC: 0.74 ± 0.083, *p* < 0.001) (Figure 3). This identified cutoff was further analyzed in a multivariate logistic regression which found that female patients with ≤ 36.6% hematocrit had significantly increased odds of medical complications (aOR: 2.815; 95% CI: 1.248–6.357; *p*=0.013).

### 3.3. Creatinine

In total, 3159 patients with creatinine were included. Within the creatinine cohort, the mean age was 59.4 years, 61.3% of patients were female, and 49.9% were obese. The mean serum creatinine value was 0.9 mg/dL (25^th^ percentile: 0.7 mg/dL, median: 0.9 mg/dL, 75^th^ percentile: 1.0 mg/dL). The average postoperative length of stay was 0.8 days (75^th^ percentile: 1 day). Upon analyzing positive outcome rates, 1.8% of patients experienced a return to the operating room, 3.2% of patients had a nonhome discharge, 1.8% of patients experienced a wound complication, 3.2% of patients experienced a medical complication, and 12.9% of patients had an eLOS.

Bivariate analysis showed higher creatinine levels in patients experiencing a medical complication (1.35 mg/dL vs. 0.92 mg/dL, *p*=0.002) and eLOS (1.06 mg/dL vs. 0.91 mg/dL, *p*=0.008). Higher creatinine levels were insignificant upon bivariate analysis for return to the operating room, nonhome discharge, and wound complications (*p* > 0.05). Multivariate analysis found that increases in creatinine heightened the odds of medical complications (aOR: 1.684; 95% CI: 1.164–2.439; *p*=0.006).

**Figure 4:**
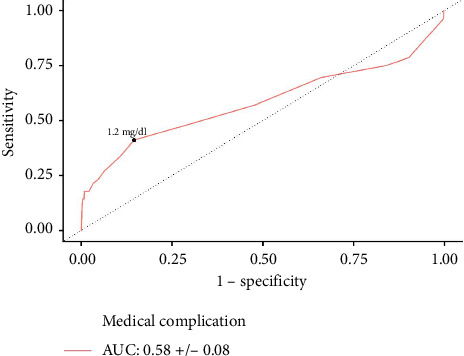
Creatinine ROC curve for predicting medical complications in male patients. A predictive creatinine threshold for medical complications was ≥ 1.2 mg/dL.

In males, AUC analysis found that ≥ 1.2 mg/dL creatinine was predictive of medical complications (AUC: 0.58 ± 0.0799, *p*=0.033) (Figure 4). This cutoff was tested in a multivariate regression, and male patients with ≥ 1.2 mg/dL creatinine had significantly increased odds of medical complications (aOR: 3.024; 95% CI: 1.093–8.370; *p*=0.033).

**Figure 5:**
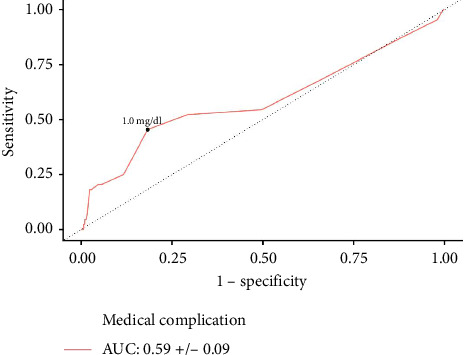
Creatinine ROC curve for predicting medical complications in female patients. A predictive creatinine threshold for medical complications was ≥ 1.0 mg/dL.

In female patients, AUC analysis found that ≥ 1.0 mg/dL creatinine was predictive of medical complications (AUC: 0.59 ± 0.089, *p*=0.039) (Figure 5). This identified cutoff was further tested in a multivariate logistic regression, which found that patients with ≥ 1.0 mg/dL creatinine had significantly increased odds of medical complications (aOR: 3.160; 95% CI: 1.234–8.088; *p*=0.016).

## 4. Discussion

Identifying determinants of short-term postoperative outcomes in hand surgery is important to promote higher levels of patient satisfaction and prevent undesirable results. Existing literature has identified age, male sex, preoperative anemia, socioeconomic deprivation, obesity, and smoking status as risk factors for complications in hand surgery [7, 12]. For instance, Hustedt et al. studied a collection of hand procedures in the NSQIP database from 2005 to 2015 and found that hypoalbuminemia, increased creatinine, and anemia were associated with postoperative complications [13]. Although previous studies have established an association between albumin, hematocrit, and creatinine with adverse outcomes in other surgical disciplines, no such study has specifically been conducted in carpal tunnel decompression patients [14–17]. Given that many cases of carpal tunnel decompression surgery are nonemergent, there is an opportunity to optimize patient comorbidities and strategically time the procedure to prevent unnecessary adverse outcomes. This study evaluated the effects of preoperative hematocrit, albumin, and creatinine on postoperative outcomes while identifying predictive thresholds in carpal tunnel decompression surgery.

Our study found that decreases in preoperative albumin and hematocrit increased the odds of medical complications, while increases in creatinine were associated with greater odds of medical complications. A cutoff of ≤ 3.5 g/dL albumin was found to be most predictive. The reference range is from 3.5 g/dL to 5.0 g/dL, and the identified cutoff of ≤ 3.5 g/dL falls on the lower bound of this range [18]. In males, a hematocrit cutoff of ≤ 39.7% was identified, which falls right below the reference range of 40%–54% in males [19]. In females, a predictive hematocrit cutoff of ≤ 36.6% was identified, which falls within the lower end of the reference range of 36%–48% for females [19]. A cutoff of ≥ 1.2 mg/dL creatinine was identified in male patients, which falls within the upper portion of the reference range, 0.7 mg/dL to 1.3 mg/dL [20]. A cutoff of ≥ 1.0 mg/dL creatinine was identified in female patients, which falls within the reference range of 0.6 mg/dL to 1.1 mg/dL [20].

The present study found that decreased albumin was associated with increased odds of medical complications. A previous study conducted by Luchetti et al. analyzed the NSQIP database for hand surgeries from 2005 to 2013 and found that hypoalbuminemia, defined by < 3.5 g/dL, was associated with higher rates of mortality along with minor and major medical complications [14]. Specifically, they reported that hypoalbuminemia was associated with outcomes, including sepsis, septic shock, deep infection, pneumonia, reintubation, and ventilator requirement > 48 h. This directly aligns with the prognostic albumin cutoff identified in our study based on ROC analysis. Another study studied patients undergoing open reduction and internal fixation for distal radius fractures in NSQIP from 2007 to 2015 and reported that preoperative hypoalbuminemia was associated with higher odds of postoperative complications, mortality, and various health resource utilization outcomes [21]. Although our analysis did not find a significant association between preoperative albumin levels and wound complications, multiple studies have found that hypoalbuminemia is associated with adverse wound outcomes [22, 23]. However, a study conducted by Karamanos et al. that studied patients undergoing body contouring procedures found no significant associations between preoperative albumin and adverse outcomes [24]. Mechanistically, albumin deficiency has been associated with decreased collagen synthesis, a prolonged inflammatory phase, and delayed epithelial proliferation, which can explain the effect of hypoalbuminemia on adverse wound outcomes [25–27].

Given that many cases of carpal tunnel decompression surgery are nonemergent, interventions can often be optimally timed. Nutritional supplementation measures can be implemented to address malnutrition in the preoperative setting as seen in the previous literature [28, 29]. Additionally, screening measures to identify at-risk patients can also be implemented using validated tools, such as the perioperative nutrition screen, which is recommended by the perioperative quality initiative [30]. Importantly, although hypoalbuminemia is commonly used as a marker of malnutrition, in inflammatory states, the liver undergoes reprioritization of protein synthesis and preferentially produces inflammatory proteins rather than albumin [31].

In our study, patients with low hematocrit were more likely to experience medical complications. Low preoperative hematocrit levels have been associated with increased postoperative morbidity in numerous surgical cohorts [9, 32]. For example, Masoomi et al. reported that in a national cohort of patients undergoing autologous breast reconstruction, preoperative anemia was associated with higher odds of postoperative complication and extended length of stay [15]. Donato et al. analyzed the NSQIP database from 2011 to 2014 for patients undergoing hand surgery and identified preoperative anemia as an independent risk factor for unplanned readmission [12]. In elective situations, steps can be taken to correct anemia based on the underlying etiology, including measures such as iron supplementation, vitamin B12 and folate supplementation, or transfusions in very severe cases [33]. Future studies should aim to understand the effectiveness of correcting anemia and weigh this against the risk of delayed surgical interventions.

The present study found that increased creatinine was associated with increased odds of medical complications. Existing studies have established an association between increased creatinine and adverse postoperative outcomes in cardiac and general surgery patients [16, 34, 35]. Within hand surgery, Zhang, Blazar, and Earp studied patients undergoing mini-open carpal tunnel release at a single institution and identified chronic kidney disease as a risk factor for complications and secondary surgery [36]. Our study found that even minor increases in creatinine were associated with increased odds of medical complications, which are corroborated by O'Brien et al., who found that modest preoperative creatinine elevations > 1.5 mg/dL were indicative of adverse outcomes in their general surgery cohort [16]. Although previous studies have established a relationship between kidney pathology and wound complications, our study did not find any associations between increased creatinine and wound complications [10]. Future research is needed to clarify best practices in optimizing kidney function amongst hand surgery patients.

Identification of these lab markers can be used to optimally time surgical interventions to prevent adverse outcomes in nonemergent scenarios. However, this delay of treatment needs to be weighed with the potential for disease progression in CTS. Damage to the median nerve is dependent on the duration and severity of compression, which supports the notion that treatment should not unnecessarily be delayed; otherwise, further median nerve damage will occur [37, 38].

Minimizing unnecessary expenses in the setting of CTS is crucial for providers as healthcare systems increasingly move to value-based models. Reoperation, nonhome discharge, and increased length of stay have been linked to increased costs in the previous orthopedic surgery literature [39–41]. Although our study did not identify any associations between the measured preoperative lab value predictor variables and health utilization outcomes, Donato et al. linked increased age, smoking, preoperative anemia, and increased operative time to the increased readmission rate in their hand surgery cohort [12]. These findings underscore the point that patient-specific risk factors can lead to increased healthcare costs, which can be avoided with optimal timing of surgical intervention.

The present study was a retrospective analysis using the NSQIP database in which the data are based on retrospective entry of patient information. This retrospective data entry enables coding error. NSQIP limits the postoperative outcome time window to 30-day time points, which may not capture outcomes that take longer to manifest; however, complications in the short term will be recorded. Furthermore, NSQIP does not record specific covariates or outcomes, which can be clinically useful in a carpal tunnel decompression cohort, such as return to work, symptom severity scales, functional status scales, electrophysiological results, and muscle weakness [42]. Furthermore, for creatinine, the identified cutoffs are likely not clinically significant as creatinine demonstrated low discriminative capabilities, reflected by an AUC close to 0.5. There may also be selection bias in terms of which patients are selected to have lab values, such as albumin, creatinine, and hematocrit drawn, especially if practitioners suspect an underlying illness. Additionally, the analysis likely systematically excluded simpler cases of carpal tunnel decompression surgery as the inclusion criteria of drawing preoperative labs would skew toward more complicated cases involving other concomitant procedures. Another consideration supporting the notion that more complex cases of carpal tunnel release were included in our sample is that the majority of patients underwent general or monitored anesthesia compared to local anesthesia. In addition, the average length of stay was one day in our cohort, which is more representative of a medically complex carpal tunnel release cohort rather than the typical carpal tunnel release patient. However, risk assessment in simple cases of carpal tunnel decompression using lab values is less applicable, whereas in more complicated cases involving carpal tunnel decompression, preoperative risk assessment is more warranted as there is a higher likelihood of adverse outcomes at baseline. It should also be noted that the CPT code for carpal tunnel decompression was never included as the primary CPT code within the NSQIP database, further supporting that the decompressions were often part of larger, more complicated surgeries.

Although using NSQIP would not account for the various outpatient surgery centers where carpal tunnel decompression can take place, only 21%–22% of cases in each cohort were conducted in the inpatient setting, and approximately 86% of cases were elective, showing that there was variability in surgical settings. Importantly, trauma cases were excluded as a part of the NSQIP data processing.

Future studies should aim to conduct similar analyses to the present investigation at a multi-institutional level. If the findings are replicated, an additional consideration for subsequent research before lab value optimization can be recommended will be to understand the cost-effectiveness involved with preoperative optimization of albumin, hematocrit, and kidney function in carpal tunnel decompression cohorts using prospective studies.

## 5. Conclusions

In this study of carpal tunnel decompression patients, decreased albumin and hematocrit were associated with greater odds of medical complications. On the other hand, increased creatinine was associated with increased odds of medical complications. Predictive cutoff values of albumin, hematocrit, and creatinine that were identified may help inform patient-provider discussions in the preoperative setting and allow for optimal timing of surgical intervention.

## Figures and Tables

**Table 1:** Cohort characteristics.

Characteristic	Albumin (*N* = 1440)	Hematocrit (*N* = 3138)	Creatinine (*N* = 3159)
Age	59.6 (14.0)^∗∗^	58.7 (14.4)^∗∗^	59.4 (14.1)^∗∗^

Average serum value	4.0 g/dL (0.5)^∗∗^	40.3% (4.7)^∗∗^	0.9 mg/dL (0.6)^∗∗^

Operative time	92.3 (72.0)^∗∗^	93.7 (72.6)^∗∗^	92.9 (70.9)^∗∗^

*Gender*			
Female	898 (62.4%)	1932 (61.6%)	1938 (61.3%)
Male	542 (37.6%)	1206 (38.4%)	1221 (38.7%)

*Race*			
American Indian/Alaska Native	11 (0.8%)	20 (0.6%)	18 (0.6%)
Asian	30 (2.1%)	54 (1.7%)	57 (1.8%)
Black	138 (9.6%)	279 (8.9%)	295 (9.3%)
Native Hawaiian or Pacific Islander	2 (0.1%)	8 (0.3%)	7 (0.2%)
White	1259 (87.4%)	2777 (88.5%)	2782 (88.1%)

*Ethnicity*			
Hispanic	175 (12.2%)	399 (12.7%)	374 (11.8%)
Not Hispanic	1265 (87.8%)	2739 (87.3%)	2785 (88.2%)

*Comorbidities*			
Current smoker	324 (22.5%)	642 (20.5%)	647 (20.5%)
Obese (BMI ≥ 30)	702 (48.8%)	1498 (47.7%)	1575 (49.9%)
Diabetes	307 (21.3%)	614 (19.6%)	683 (21.6%)
Dyspnea symptoms	82 (5.7%)	170 (5.4%)	181 (5.7%)
Hypertension requiring medication	807 (56.0%)	1590 (50.7%)	1719 (54.4%)
COPD	82 (5.7%)	159 (5.1%)	168 (5.3%)
Renal failure	2 (0.1%)	3 (0.1%)	3 (0.1%)
Currently on dialysis	16 (1.1%)	21 (0.6%)	21 (0.7%)
Malnourishment	5 (0.3%)	9 (0.3%)	9 (0.3%)
Bleeding disorder	74 (5.1%)	131 (4.2%)	138 (4.4%)
CHF	7 (0.5%)	13 (0.4%)	15 (0.5%)
Systemic sepsis	56 (3.9%)	91 (2.9%)	94 (3.0%)
Disseminated cancer	3 (0.2%)	3 (0.1%)	3 (0.1%)
Ascites	1 (0.07%)	1 (0.03%)	1 (0.03%)
Ventilator dependent	2 (0.1%)	2 (0.06%)	2 (0.06%)
Open wound/wound infection	52 (3.6%)	91 (2.9%)	90 (2.8%)
Immunosuppressive therapy	65 (4.5%)	123 (3.9%)	122 (3.9%)

*Functional status*			
Independent	1410 (97.9%)	3090 (98.5%)	3114 (99.5%)
Partially dependent	26 (1.8%)	42 (1.3%)	38 (1.2%)
Totally dependent	4 (0.3%)	6 (0.2%)	7 (0.2%)

*Surgical specialty*			
Cardiac surgery	1 (0.07%)	2 (0.06%)	2 (0.06%)
General surgery	58 (4.0%)	96 (3.1%)	90 (2.8%)
Gynecology	4 (0.3%)	13 (0.4%)	8 (0.3%)
Neurological surgery	109 (7.6%)	309 (9.8%)	296 (9.4%)
Obstetrics	1 (0.07%)	1 (0.03%)	1 (0.03%)
Orthopedic surgery	1083 (75.2%)	2318 (73.9%)	2365 (74.9%)
Otolaryngology	1 (0.03%)	3 (0.1%)	4 (0.1%)
Plastic surgery	165 (11.5%)	369 (11.8%)	367 (11.6%)
Urology	2 (0.1%)	2 (0.06%)	2 (0.06%)
Vascular surgery	16 (1.1%)	25 (0.8%)	24 (0.8%)

*Surgery type*			
Converted	5 (0.3%)	10 (0.3%)	10 (0.3%)
Endoscopic	174 (12.1%)	396 (12.6%)	399 (12.6%)
Open	1261 (87.6%)	2732 (87.1%)	2750 (87.1%)
Preoperative transfusion of ≥ 1 unit of whole/packed RBCs in 72 h prior to surgery	6 (0.4%)	7 (0.2%)	7 (0.2%)

*ASA class*			
Class 1	38 (2.6%)	155 (4.9%)	123 (3.9%)
Class 2	657 (45.6%)	1513 (48.2%)	1484 (47.0%)
Class 3	677 (47.0%)	1367 (43.6%)	1445 (45.7%)
Class 4	68 (4.7%)	103 (3.3%)	107 (3.4%)

*Anesthesia*			
Epidural	—	1 (0.03%)	1 (0.03%)
Local anesthesia	4 (0.3%)	12 (0.4%)	11 (0.3%)
General anesthesia	1073 (74.5%)	2383 (75.9%)	2359 (74.7%)
Monitored anesthesia	262 (18.2%)	493 (15.7%)	514 (16.3%)
Regional anesthesia	95 (6.6%)	237 (7.6%)	261 (8.3%)
Spinal anesthesia	3 (0.2%)	7 (0.2%)	6 (0.2%)
Other	3 (0.2%)	5 (0.2%)	6 (0.2%)

*Wound class*			
Class 1	1316 (91.4%)	2922 (93.1%)	2950 (93.4%)
Class 2	43 (3.0%)	71 (2.3%)	64 (2.0%)
Class 3	24 (1.7%)	46 (1.5%)	44 (1.4%)
Class 4	57 (4.0%)	99 (3.2%)	101 (3.2%)
Inpatient status	319 (22.2%)	695 (22.1%)	672 (21.3%)

*Surgery urgency*			
Elective	1247 (86.6%)	2722 (86.7%)	2746 (86.9%)
Emergency	98 (6.8%)	188 (6.0%)	185 (5.9%)

*Transfer history*			
Acute care hospital	13 (0.9%)	3 (0.7%)	23 (0.7%)
Admitted from the home	1398 (97.1%)	3053 (97.3%)	3074 (97.3%)
Chronic care facility	5 (0.3%)	8 (0.3%)	9 (0.3%)
Emergency department	23 (1.6%)	48 (1.5%)	47 (1.5%)
Other	1 (0.07%)	6 (0.2%)	6 (0.2%)
Nonhome discharge	61 (4.2%)	102 (3.3%)	101 (3.2%)
Postoperative length of stay	1.0 (3.2)⁣^∗∗^	0.8 (2.6)⁣^∗∗^	0.8 (2.5)⁣^∗∗^
Return to the operating room	29 (2.0%)	61 (1.9%)	58 (1.8%)
Any wound complication	28 (1.9%)	56 (1.8%)	56 (1.8%)
Any medical complication	67 (4.7%)	101 (3.2%)	100 (3.2%)
Extended length of stay	221 (15.3%)	421 (13.4%)	409 (12.9%)

Note: Values shown as *N* (%).

Abbreviations: ASA, American Society of Anesthesiologists; CHF, congestive heart failure; COPD, chronic obstructive pulmonary disease; RBCs, red blood cells.

⁣^∗∗^Mean (standard deviation).

**Table 2:** Bivariate and multivariate results.

Outcome variable	Albumin			Hematocrit			Creatinine		
	Bivariate	Multivariate		Bivariate	Multivariate		Bivariate	Multivariate	
	*p* value	Adjusted OR (95% CI)	*p* value	*p* value	Adjusted OR (95% CI)	*p* value	*p* value	Adjusted OR (95% CI)	*p* value
Return to the operating room	0.047⁣^∗^	0.981 (0.367, 2.623)	0.969	0.476	—	—	0.440	—	—
Nonhome discharge	< 0.001⁣^∗^	1.130 (0.567, 2.249)	0.729	< 0.001⁣^∗^	0.951 (0.903, 1.000)	0.051	0.179	—	—
Any wound complication	0.150	—	—	0.115	—	—	0.406	—	—
Any medical complication	< 0.001⁣^∗^	0.479 (0.242, 0.948)	**0.035**⁣^∗^	< 0.001⁣^∗^	0.889 (0.846, 0.934)	**< 0.001**⁣^∗^	0.002⁣^∗^	1.684 (1.164, 2.439)	**0.006**⁣^∗^
Extended length of stay	< 0.001⁣^∗^	0.580 (0.331, 1.016)	0.057	< 0.001⁣^∗^	0.992 (0.955, 1.030)	0.673	0.008⁣^∗^	1.268 (0.867, 1.854)	0.221

*Note:* The bold values signify statistically significant *p* values in the multivariate logistic regression analysis.

Abbreviations: CI, confidence interval; OR, odds ratio.

⁣^∗^Statistical significance as defined by *p* < 0.05.

**Table 3:** Receiver operating characteristic curve cutoff analysis results for medical complications.

**Preoperative lab**			

**Albumin**			
**Cutoff**	**AUC ± 95% CI**	**p** **value**	**Sensitivity, specificity**

≤ 3.5 g/dL	0.79 ± 0.065	< 0.001⁣^∗^	61.2%, 87.6%

**Hematocrit—male**			
**Cutoff**	**AUC ± 95% CI**	**p** **value**	**Sensitivity, specificity**

≤ 39.7%	0.77 ± 0.074	< 0.001⁣^∗^	70.9%, 79.8%

**Hematocrit–female**			
**Cutoff**	**AUC ± 95% CI**	**p** **value**	**Sensitivity, specificity**

≤ 36.6%	0.74 ± 0.083	< 0.001⁣^∗^	65.2%, 76.7%

**Creatinine—male**			
**Cutoff**	**AUC ± 95% CI**	**p** **value**	**Sensitivity, specificity**

≥ 1.2 mg/dL	0.58 ± 0.079	0.033⁣^∗^	41.1%, 85.5%

**Creatinine—female**			
**Cutoff**	**AUC ± 95% CI**	**p** **value**	**Sensitivity, specificity**

≥ 1.0 mg/dL	0.59 ± 0.089	0.039⁣^∗^	45.5%, 81.7%

Abbreviations: AUC, area under the ROC curve; CI, confidence interval.

⁣^∗^Statistical significance as defined by *p* < 0.05.

## Data Availability

The data that support the findings of this study are derived from a publicly available database.

## Supporting Information

Supporting Table 1: This table outlines the patient characteristics included in the multivariate logistic regression analyses.
