# Supplementary material for: Exploring the Impact of Preoperative Laboratory Values on Short-Term Outcomes in Complex Carpal Tunnel Decompression Surgery
Source: Adv Orthop. 2025 Jan 22;2025:8494043. doi: 10.1155/aort/8494043 (PMC11779986; doi:10.1155/aort/8494043)
Supplement: Supporting Information — Additional supporting information can be found online in the Supporting Information section. [file 8494043.f1.docx]

**Supplemental Table 1. Variables Included in Multivariate Logistic Regression.**

| **Variable Name** |
| --- |
| Age |
| Gender |
| Race |
| Hispanic Ethnicity |
| Operative Time |
| Surgical Specialty |
| Surgery Type |
| Inpatient/Outpatient |
| Obesity |
| Diabetes |
| Dyspnea |
| Functional Health Status |
| Ascites |
| Heart Failure in 30 days before surgery |
| Hypertension requiring medication |
| Acute renal failure |
| History of Severe COPD |
| Currently on Dialysis |
| Open Wound/Wound Infection |
| Immunosuppressive therapy |
| Malnourishment |
| Bleeding Disorders |
| Current Smoker within One Year |
| Preoperative Transfusion of ≥1 unit of whole/packed RBCs in 72 hours prior to surgery |
| ASA classification |
| Ventilator dependent |
| Systemic sepsis |
| Disseminated cancer |
| Transfer status |
| Elective surgery |
| Emergency case |
| Wound classification |
| Days from Hospital Admission to Operation |
| Principal Anesthesia Technique |
